# Bahir Dar Child Development Cross-Sectional Study, Ethiopia: study protocol

**DOI:** 10.1136/bmjpo-2024-003173

**Published:** 2025-04-02

**Authors:** Sarah K G Jensen, Kalkidan Yibeltal, Krysten North, Firehiwot Workneh, Atsede Teklehaimanot, Betelhem Haimanot Abate, Nebiyou Fasil, Tizita Lemma Melka, Theresa I Chin, Lian V Folger, Unmesha Roy Paladhi, Fred Van Dyk, Moriah E Thomason, Patricia Ellen Grant, Terrie Inder, Alemayehu Worku, Yemane Berhane, Anne CC Lee

**Affiliations:** 1 Developmental Medicine, Boston Children’s Hospital, Boston, Massachusetts, USA; 2 Harvard Medical School, Boston, Massachusetts, USA; 3 Department of Reproductive Health and Population, Addis Continental Institute of Public Health, Addis Ababa, Ethiopia; 4 Department of Pediatric Medicine, Brigham and Women’s Hospital, Boston, Massachusetts, USA; 5 Department of Epidemiology and Biostatistics, Addis Continental Institute of Public Health, Addis Ababa, Ethiopia; 6 Pediatrics and Child Health, College of Health Science Tikur Anbesa Specialized Hospital, Addis Ababa, Ethiopia; 7 Addis Continental Institute of Public Health, Addis Ababa, Ethiopia; 8 Global Health and Health Policy, Addis Continental Institute of Public Health, Addis Ababa, Ethiopia; 9 Department of Psychology, Bahir Dar University, Bahir Dar, Ethiopia; 10 Department of Pediatrics, Warren Alpert Medical School of Brown University, Providence, Rhode Island, USA; 11 Department of International Health, Johns Hopkins Bloomberg School of Public Health, Baltimore, Maryland, USA; 12 Department of Child and Adolescent Psychiatry, New York University Grossman School of Medicine, New York, New York, USA; 13 Boston Children’s Hospital, Boston, Massachusetts, USA; 14 Children’s Hospital of Orange County, Orange, California, USA

**Keywords:** Neurodevelopment, Low and Middle Income Countries

## Abstract

**Introduction:**

Foundational preacademic skills are crucial for academic success and serve as predictors of socioeconomic status, income and access to healthcare. However, there is a gap in our understanding of neurodevelopmental patterns underlying preacademic skills in children across low-income and middle-income countries (LMICs). It is essential to identify primary global and regional factors that drive children’s neurodevelopment in LMICs. This study aims to characterise the typical development of healthy children and factors that influence child development in Bahir Dar, Ethiopia.

**Methods and analysis:**

The Bahir Dar Child Development Study is a cross-sectional study implemented in two health centres, Shimbit and Abaymado and in Felege Hiwot Comprehensive Specialized Hospital (FHCSH) in Bahir Dar, Amhara, Ethiopia. Healthy children between 6 and 60 months of age will be recruited from the health centres during vaccination visits or via community outreach. Young children aged 6–36 months will complete the Global Scale for Early Development. A battery of paper and tablet-based assessments of neurocognitive outcomes including visual and verbal reasoning, executive functions and school readiness will be completed for children aged 48–60 months. Caregivers will respond to surveys covering sociodemographic information, the child’s medical history and nutrition, and psychosocial experiences including parental stress and mental health. During a second visit, participants will undergo a low-field MRI scan using the ultra-low-field point-of-care Hyperfine MRI machine at FHCSH. Analyses will examine relationships between risk and protective factors, brain volumes and neurocognitive/developmental outcomes.

**Ethics and dissemination:**

The study is approved by the Institutional Review Boards of Addis Continental Institute of Public Health (ACIPH/lRERC/004/2023/Al/05-2024), Mass General Brigham Hospital (2022P002539) and Brown University (STUDY00000474). Findings will be disseminated via local dissemination events, international conferences and publications.

**Trial Registeration number:**

NCT06648863.

**WHAT IS ALREADY KNOWN ON THIS TOPIC**

- The rapid rate of neurodevelopmental growth early in life leaves the brain susceptible to environmental influences, including adverse effects of biological and psychosocial hazards, such as malnutrition, infectious disease, parental stress and depression, and understimulating environments. Early life also presents a window of opportunity, however, where early interventions may be most effective.

**WHAT THIS STUDY ADDS**

- This study contributes to the growing body of knowledge regarding brain and neurocognitive development in children in low-resource environments, who have historically been under-represented in research.
- We will characterise patterns of neurodevelopment in infants and children aged 6–60 months, and examine environmental risks and protective factors that may predict neuroanatomical outcomes in children in Ethiopia.
- We will generate evidence of associations between objective markers of neural anatomy and child neurocognitive development in children in Ethiopia.
- We will generate experience to guide the implementation of new technologies and tools to objectively assess neurodevelopment in young children in low resource environments in low and middle-income countries.

## Introduction

 Worldwide, more than 250 million children under 5 years of age fail to meet their developmental potential due to risk factors associated with poverty and food insecurity.^1^ In Ethiopia, 88% of children are exposed to multidimensional poverty, defined as the unfulfilment of rights or needs for basic goods and services, which are inherent risk factors for poor neurodevelopment.^2^ Reduced or impaired neurocognitive development early in life can impact lifelong patterns of cognitive and behavioural development, beginning with early, preacademic skills that are vital to academic success and predictive of later health and socioeconomic standing.^3^ A diverse array of environmental factors that are widespread in low-resource communities can shape children’s neurological and cognitive development. Beginning in fetal development, biological factors such as maternal nutrition, infection and stress can impact fetal neurological development and growth with long-lasting impacts throughout development. Postnatally, maternal and infant nutrition, exposure to disease, environmental neurotoxins, maternal mental health, and parental engagement in psychosocial interaction and stimulation of the infant are some of the factors known to influence developing brain systems and impact emerging cognitive and behavioural functions in children.^4 5^ Most evidence to date comes from Western, industrialised countries, leaving a gap in our understanding of neurodevelopmental patterns in children across low-income and middle-income countries (LMICs) where neurodevelopmentally impactful adversities tend to be magnified. Increased knowledge of risk and protective factors associated with neurodevelopmental characteristics in children can guide targeted interventions to address risk factors that drive poor neurodevelopmental outcomes in children growing up in low-resource environments.^6^

The Bahir Dar Child Development (BCD) study will characterise patterns of typical neurodevelopment in a cross-sectional community sample of children in Bahir Dar, Ethiopia. Data will be harmonised with similar data collected from other countries to provide a global map of child development and identify important targetable factors that will improve neurodevelopmental outcomes in children. The first goal of the study is to examine correlations between brain volumes and neurocognitive/developmental outcomes. The second goal is to examine associations between risk and protective factors and neurodevelopmental outcomes. We will explore these relationships across different age groups. We focus on developmental and neurocognitive outcomes that reflect early developmental milestones in children aged 6–36 months. In older children aged 48 and 60 months, we assess domains that we believe contribute to school readiness, including aspects of visual reasoning, attention, processing speed, verbal development and early literacy, numeracy and early knowledge. Previous studies in LMIC have shown differences in associations of early life exposures and neurodevelopmental outcomes.^7^

## Methods and analysis

### Study design

The BCD Cohort is a cross-sectional observational study designed to characterise typical neurodevelopment in the first 5 years of life in Bahir Dar, Ethiopia.

### Study setting

Bahir Dar is the capital city of the Amhara Region in Ethiopia where the official language is Amharic. The population of Bahir Dar was projected to be 348 429 in 2017.^8^ According to the 2023 United Nations Development Programme multidimensional poverty index, 27% of the Ethiopian population lives below the US$2.15 per day income poverty line.^9^ According to the 2019 Ethiopian Demographic and Health Survey report, 49.8% of women aged 15–49 years in the Amhara region are literate.^10^ Moreover, the rate of stunting among children under 5 years of age is 41.5%.^10^ The study will be conducted in two health centres in Bahir Dar (Shimbit and Abay Mado) and Felege Hiwot Comprehensive Specialized Hospital (FHCSH). FHCSH is the main referral hospital in the region and delivers healthcare services for more than 10 million people in Amhara and neighbouring regions. A Hyperfine low-field (0.064 Tesla) MRI machine was placed in FHCSH on 18 June 2022 and the installation was completed on 1 September 2022.

### Patient and public involvement

Before starting the project, we conducted formative research via key informant interviews with community members, partners, in-laws, religious/community leaders and other relevant stakeholders to develop a community-guided research strategy for introducing low-field MRI in our research. These efforts informed the design and development of educational material for study participants, including videos and the community sensitisation approach. We also consulted with local psychologists, physicians and childcare professionals when developing and testing the assessment battery.

### Study participants and recruitment

The BCD study will enrol healthy children from age 6 to 60 months across eight age cohorts (see table 1). Eligible children will be identified during routine outpatient and vaccination visits at the study health centres. Children who present with clinical symptoms based on parental/child self-report will not be eligible to participate. We will aim to recruit N=50 children into each cohort of the eight cohorts for a total cross-sectional sample size of N=400. Inclusion and exclusion criteria and additional MRI screening questions are shown in table 2.

**Table 1:** Overview of data collection across the cross-sectional cohorts (A–H)

	Enrolment visit		MRI visit
**Cohort**	Assessments	Surveys	Assessments
A/6 monthsB/12 monthsC/18 monthsD/24 monthsE/30 monthsF/36 months	GSED long form (secondary outcome) GSED short form (secondary outcome) Anthropometrics Haemoglobin	Sociodemographic information Home environment Parenting Health history	MRI (Primary outcome)
G/48 monthsH/60 months	Neurocognitive development assessment of reasoning and verbal expression (secondary outcome) Executive functioning (secondary outcome) School readiness (secondary outcome) Anthropometrics Haemoglobin 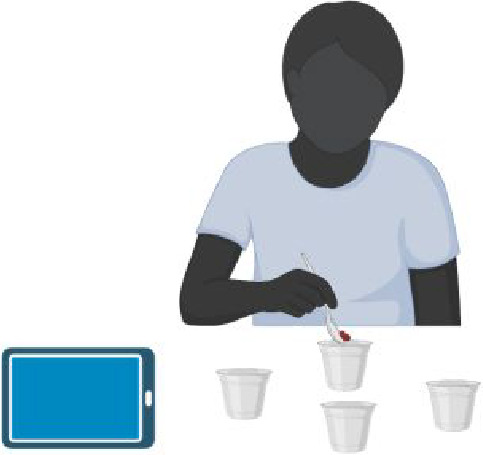	Sociodemographic information Home environment Parenting Health history 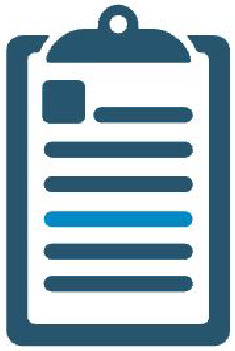	MRI (Primary outcome) 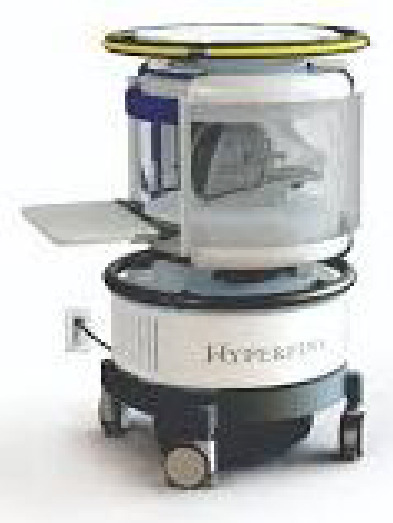

GSEDGlobal Scale for Early Development

**Table 2:** Inclusion and Exclusion criteria

Inclusion criteria	Exclusion criteria
Child lives in Bahir Dar with parents who are willing to travel to FHCSH clinic for the MRI. Family has no plans to move outside of the catchment area in the next 6 months Child is healthy without active clinical symptoms/complaints. The child is within specified age bins for one of the cohort/group±1 months for the 6, 12 and 18 months old and ±2 months for the 24, 36, 48 and 60 months old children.	Child was born with a congenital birth defect or hypoxic ischaemic encephalopathy (birth asphyxia) per maternal report. Child has developmental delay or behavioural disorder per maternal report. Child has clinical signs or symptoms of illness based on maternal and child report (including vomiting, headache or seizures).
Additional screening for MRI scanning	
	Surgical hardware such as staples or screws Metal shrapnel or bullets; Suspected metal in eye; metal jewellery or watch; Metal-containing tattoos or permanent make-up on head or neck.

FHCSHFelege Hiwot Comprehensive Specialized Hospital

### Sample size calculation

The sample size of n=50 children per cohort was selected to ensure collection of at least 30 high-quality MRI per cohort. Expected reasons for data loss include loss to follow-up since MRI is collected during a second visit and poor quality of the images due to movement artefact or incomplete brain visualisation. MRI is an objective quantitative measure; this sample size is widely considered adequate to establish normal population distributions (mean, SD) of brain parameters and brain volumes.^11^

### Study procedures and data collection

#### Enrolment visit and behavioural assessment

Immediately after the caregiver provides informed consent, the enrolled child will proceed to complete the first visit. Children will undergo a neurocognitive and developmental assessment outlined in and mothers will complete surveys to characterise family sociodemographics, home environment, recent events and hardships, maternal mental health, and child health and nutrition. The protocol for the neurocognitive and developmental assessment varies by age to ensure the use of age-appropriate assessment tools as outlined in table 1. We will also do a finger prick blood test to measure haemoglobin levels.

### MRI visit

Families will travel to FHCSH within 1 month of the enrolment visit to complete the MRI scans.

## Measures

Data will be collected via the Survey Solutions platform (World Bank, V.20.08, 2021). Study nurses enter data directly into electronic tablets with programmed validity checks during study visits. The tablets are regularly synchronised to a server on the ACIPH campus.

## MRI

MRI imaging protocol: MRI data collection will be performed by a trained radiography technologist using the portable, ultra-low field (0.064 Tesla) Hyperfine MRI system (Guilford, Connecticut, USA) installed at FHCSH. We will use the UNITY consortium common protocol which consists of volumetric T1 and T2-weighted imaging and quantitative T2 imaging, optimised for infant/paediatric brain (table 3).^12^ Magnetisation-prepared rapid gradient echo sequences will be used for volumetric T1 imaging, and turbo-spin echo acquisition will be used to attain T2-weighted images.

**Table 3:** The main imaging protocol used in the UNITY studies for children older than 1 month

	Image matrix (X×Y×Z)	Resolution(X×Y×Z) mm^3^	TE/TR/TI (ms)	Time (min:s)
T2 FSE (axial)	112×136×40	1.5×1.5×5	180/2000/NA	2:15
T2 FSE (coronal)	112×44×124	1.5×1.5×5	220/2000/NA	2.22
T2 FSE (sagittal)	36×136×124	1.5×1.5×5	225/2000/NA	2:12
T1 IR-FSE (axial)	112×138×40	1.5×1.5×5	6.6/880/354	6:11
T2 Mapping (optional)	20×18×22	1.7×1.7×5	41, 81, 122, 163, 204, 244, 285, 326, 366, 407/2000/NA	9:22

FSEFast Spin EchoTEEcho timeTIInversion timeTRRepetition time

The protocol for the MRI study visit was developed based on formative work conducted before the study as part of the Longitudinal Infant Development and Growth (LIDG) study.^13^ During the scan, caregivers will be invited to stay in the scanning room to provide comfort and encouragement as needed. Appointments will be scheduled during nap times or when children are anticipated to be tired. Infants and young children will be fed, soothed to sleep and placed in the scanner. Older children who remain awake during the scan will be encouraged to lie still, and a tablet may be used immediately outside the scanner as a distraction. Earplugs will be placed prior to scanning to prevent startling or awakening during the scan. The sequences take around 35 min to complete. Scans will be repeated up to two times if movement or image artefacts are significant during live monitoring of the scan quality. A clinical read/interpretation of the MRI will be done by senior radiologists at FHCSH within 1 week of the scan. Incidental findings will be communicated to the family along with a plan for referral. The primary MRI outcome will be total supratentorial brain volume. We will also assess ventricular, Cerebrospinal Fluid (CSF), and thalamus volumes.

### Other measures

All children will undergo an anthropometric assessment and a cognitive developmental assessment. Children aged 6–36 months will complete the WHO Global Scales for Early Development (GSED) while children aged 48 and 60 months will complete a battery of tasks selected to assess different aspects of cognitive development, executive functions and self-regulation as well as a tool to assess early maths skills, literacy and knowledge as all of these domains are believed to reflect aspects of child-level school readiness. Tools were piloted in March 2024, and minor adaptations were made to be culturally relevant, feasible, acceptable and age appropriate.

#### Child anthropometrics assessment

Anthropometric assessments will be done at the enrolment visit using standard methods described by INTERGROWTH-21.^14^ Infant weight will be measured using a high-quality digital scale (ADE M112600, Germany; precision 5 g). Child weight will be measured with a high-quality digital scale (ADE M317600, Germany; precision 100 g). For infants under 24 months of age, we will measure recumbent length to the mm using a portable infantometer (Perspective Enterprises PE-RILB-LTWT, Michigan USA, precision 1 mm), which has a fixed headboard and movable footboard. For children aged 24 months and older, height will be measured using a high-quality adult stadiometer (Shorr Productions). Head and mid-upper arm circumferences will be measured to the nearest millimetre using insertion tapes (Shorr Productions). Regular calibration checks will be made to ensure the accuracy of the scales. Parameters will be measured in duplicate.

#### WHO Global Scales for Early Development

The Global Scales for Early Development (GSED) was developed by a team assembled by the World Health Organization (WHO) to assess global child development across cultures and settings in children aged 0–36 months of age and will be used to assess child development in children aged 6–36 months.^15^ The GSED assesses development across multiple domains including cognition, motor, language and socioemotional development. We will use both the short form, a caregiver report and the long form, a direct assessment which can be used to derive a global D-score for overall child development. The GSED has been validated in Tanzania, Bangladesh and Pakistan,^16^ and more validations are underway.^17^ We worked with WHO to translate, culturally adapt and field-test the instruments for the Ethiopian context. The adaptation process followed a process whereby it was translated into Amharic language by two independent early childhood experts who held a consensus meeting with local early child development field experts to make sure the tool was culturally appropriate. These adaptations were then back-translated, then approved by WHO and piloted. Two Ethiopian master trainers (KY and AT) were trained by WHO and trained a team of local study nurses. The GSED takes about 1 hour to administer and uses the Open Data Kit platform.

#### Visual reasoning, verbal expression and school readiness

We will use paper-based tasks to assess visual reasoning, verbal expression and school readiness in children aged 48–60 months. These domains were selected as important to school readiness as reflecting preacademic knowledge and mental flexibility and preparedness to learn. These tasks were also selected because they had been used previously with children in a similar age range in Bahir Dar.^18^ We did further piloting and made minor adaptations based on feedback from children and experts during prestudy piloting. Changes concerned drawn images that the child did not recognise; for example, for a lake picture, we selected higher quality more recognisable images. These tasks take about 15 min to complete.

Visual matrix reasoning: Children will be shown stimuli in a booklet containing matrix diagrams and asked to choose an image they think will complete a sequence of 4–5 images. The task has 3 practice items and 29 test items. The complexity of the task increases as the task progresses.

Verbal expression: An assessor will read a sentence similar to ‘X and Y are both …’, X and Y being two objects or phenomena that represent a shared category. The child completes the sentence by stating what X and Y both represent, for example, ‘blue and green are both colours’. The task has 3 practice items and 20 test items. Concepts increase in complexity as the task progresses. The child’s verbatim response is written down for each item.

School readiness: A battery of verbal and paper and pen-based tasks that test concepts relevant to early knowledge formation and important skills for success in school, including knowledge of literacy, numeracy, early reading, crystallised knowledge and working memory will be used to assess preacademic knowledge.

#### Executive functions

Executive functions will be assessed in children ages 4–5 using three tasks from the National Institute of Health (NIH) Toolbox listed below. Tasks were selected to reflect different aspects of executive functions such as attention, inhibitory control and processing speed which we believe are important for learning and readiness for school. The NIH Toolbox App is administered on an iPad (iOS 16) and the assessment time is around 20 min. Computer-generated scores reflect accuracy and reaction time metrics.^19^ Children are asked to go as fast as they can without making mistakes.

Dimensional Change Card Sort (DCCS): DCCS measures cognitive flexibility and attention. Children are shown figures (stimuli) that vary across two dimensions, shape and colour. The child is asked to select one of two stimuli that best match a central target stimulus based on one characteristic (shape or colour). Children practise matching based on colour and shape before the actual trial items

Flanker Inhibitory Control and Attention Task: The Flanker Task measures inhibitory control and attention. Children are presented with a row of fish and/or arrows and have to select a response button that shows the direction of the central stimulus (fish or arrow) by pressing one of two buttons on the screen.

Speeded matching: The Speed matching task assesses processing speed. Children are asked to identify which of four targets (pictures of two-dimensional animal faces) match the target picture at the top of the screen by tapping an image as quickly as possible without making mistakes. The task starts with four practice items with feedback. The trial finished after 90 s.

#### Self-regulation

In addition to cognitive skills and early knowledge, self-regulation is an important aspect of school readiness as it helps children learn and thrive in a classroom setting and in social interactions. Study nurses will complete a brief survey assessing the child’s observed self-regulation skills in children aged 48–60 months. This survey was adapted from items from The International Development and Early Learning Assessment (IDELA)^20^ and the Preschool Self-Regulation Assessment^21^ to be culturally relevant and age appropriate. It assesses behaviours such as attention, confidence in own abilities, diligence, frustration and positive mood. The eight items are scored on a 4-point Likert scale and summed to create a total score.

#### Surveys

Surveys completed by the mother at the enrolment visit are listed in box 1.

Box 1 **Outcome measures**

**Primary outcome**

- Total supratentorial brain volume.

**Secondary outcomes**

- Global child development (Global Scale for Early Development scores) (6–36 months).
- Cognitive functioning (48–60 months).
- School readiness (48–60 months).
- Executive functioning (48–60 months).
- Self-regulation (48–60 months).

**Surveys on key exposures (assessed by maternal self-report)**

- Maternal education, occupation, self-reported ranking of socioeconomic status and a list of assets and housing conditions used to create a socioeconomic index.
- Maternal depression and perceived stress are assessed using the 9-item Patient Health Questionnaire and the 10-item Cohen’s Perceived Stress Scale, both of which were previously validated in Ethiopia.^22^
- Child health and morbidity questions include reports of birth complications, hospitalisations since birth, serious medical conditions, chronic conditions and acute illness in the last 12 months including febrile illness, diarrhoea, malaria, respiratory illness and child emotional well-being (eg, depression/tearfulness, fears and easily scared).
- Childhood Adversity Index assesses using 13 items adapted from the Daily Hardships Survey.
- Child diet questionnaires, including breastfeeding history, dietary diversity, nutrient intake from 7-day semiquantitative food frequency questionnaires, food insecurity.
- Parenting/family environment (caregiver engagement in stimulating activities and learning materials in the home assessed using items from the UNICEF Multiple Cluster Indicator Survey).

**Other exposures (measured by research staff)**

- Anthropometrics (height-for-age, weight-for-age and weight-for-height).
- Child haemoglobin/anaemia (measured at study visit).

### Quality control

Quality assurance checklists will ensure that measurements meet a minimal standard. Staff will be observed and required to achieve competency before conducting neurobehavioural and developmental assessments. We will monitor the quality of data by reviewing 10% video-recorded assessments of the GSED assessments. MRI will be reviewed by the MRI technician during acquisition and further reviewed by local radiologists and researchers at Brown University for quality control and to provide feedback to the MRI technician.

## Analysis

### Statistical analysis

Descriptive statistics (eg, means and SD, frequencies, and percentages) for key variables will be examined for each cohort. We will use a linear regression approach to estimate associations between exposures and continuous outcomes overall and by cohort. Regression assumptions will be checked and methods such as natural log (ln) transformation may be used to meet normality assumptions. Where appropriate, we will standardise data using relevant reference samples or z-scores created within the sample. We will account for key confounders using a multivariable approach. Baseline characteristics associated with both the outcome and exposure status (p<0.10) will be included in adjusted models as potential confounders.

### Confidentiality

Data are kept confidential on a local encrypted server. Enrolment and consent forms are the only study documents where the study participant’s name will be recorded. Paper copies of data forms will be stored in a locked file when not in use. Access to data files containing personal identifying information will be limited to the principal investigators and key staff.

## Dissemination plan

Study findings will be disseminated to local and global stakeholders and research communities including the Ethiopian Ministry of Health and zonal health departments. Involving relevant stakeholders in the dissemination process will enhance awareness of developmental trends in children’s neurocognitive development and support the integration of key findings into local programmes supporting families with young children. Modes of dissemination will include reports and presentations. Abstracts will be submitted to local and global conferences.

## Conclusions

By cross-sectionally assessing children across seven age categories, we will map stages of neurological development in a semi-rural population in sub-Saharan Africa. The study was designed to be harmonised with similar ongoing cohort studies collecting MRI in paediatric populations globally.^12^ The combined assessment of neurocognitive and MRI outcomes will allow us to examine possible associations between neuroanatomical outcomes and neurocognition. Using questionnaires to characterise children’s biological and psychosocial experiences, we will be able to assess associations between neurocognitive outcomes and environmental experiences. Analyses will examine correlations between neuroanatomical outcomes and cognitive/behavioural outcomes as well as associations of adverse risk factors (eg, low socioeconomic status and poverty, food insecurity, stunted growth, poor maternal mental health, high levels of hardship) and protective factors (eg, stimulating home environments) with neuroanatomical and cognitive–behavioural outcomes. Few studies have implemented MRI in semi-rural settings in LMICs. This study will generate data to guide studies and future interventions for traditionally hard-to-reach and under-represented research populations.

## Data Availability

Data are available on reasonable request.
