# Supplementary material for: Bahir Dar Child Development Cross-Sectional Study, Ethiopia: study protocol
Source: BMJ Paediatr Open. 2025 Apr 2;9(1):e003173. doi: 10.1136/bmjpo-2024-003173 (PMC11969594; doi:10.1136/bmjpo-2024-003173)
Supplement: Supplementary file 1 [file bmjpo-2024-003173.reviewer_comments.pdf]

PEER REVIEW HISTORY

BMJ Paediatrics Open publishes all reviews undertaken for accepted manuscripts. Reviewers are asked to complete a checklist review form and are provided with free text boxes to elaborate on their assessment. These free text comments are reproduced below.

ARTICLE DETAILS

**TITLE (PROVISIONAL)**	Bahir Dar Child Development Cross-sectional study, Ethiopia: Study Protocol
**AUTHORS**	Jensen, Sarah K. G.; Yibeltal, Kalkidan; North, Krysten; Workneh, Firehiwot; Teklehaimanot, Atsede; Abate, Betelhem Haimanot; Fasil, Nebiyou; Melka, Tizita Lemma; Chin, Theresa I.; Folger, Lian V.; Roy Paladhi, Unmesha; Van Dyk, Fred; Thomason, Moriah E.; Grant, P. Ellen; Inder, Terrie; Worku, Alemayehu; Berhane, Yemane; Lee, Anne

VERSION 1 - REVIEW

**REVIEWER NAME**	***Taryn Smith***
**REVIEWER AFFILIATION**	University of Liverpool
**REVIEWER CONFLICT OF INTEREST**	N/A
**DATE REVIEW RETURNED**	21-Nov-2024

**GENERAL COMMENTS**	This protocol describes the Bahirdar Child Development study, a cross-sectional study aiming to characterise the brain and cognitive development of typically developing children spanning 6 – 60 months and factors influencing this development. This study will address gaps in our understanding of biological, environmental and social factors that influence child brain and cognitive development in resource-limited contexts. The authors have clearly described the rationale for the study and data collection methods. Findings from this study are likely to provide insights into a range of factors which influence brain development across a developmental stage of significant brain development (first 5 years) and may point to target and time-sensitive interventions to optimise child development.<br><br>Some minor comments and suggestions:<br><br>1. In the Study Setting it would be useful to add more information on the setting and the general population of Bahirdar to put the study into context. Similarly, how many language groups are present in the study area and how will this be considered in study recruitment and data collection?<br><br>2. Table 2, final bullet point under the inclusion criteria, is there some age bands 24 – 60 months) missing at the end of the sentence?<br><br>3. A rationale for why the specific child development measures (visual reasoning, verbal expression, school readiness, executive functions) were selected should be briefly added.

	4. The authors state that adaptations to the child development tools have been made. It would be helpful to briefly describe these adaptations.<br><br>5. I find the surveys for the key exposures inadequately described (Table 4). For example, PHQ-9 is neither defined nor described. How is nutrient intake being estimated? Or is it simply dietary diversity that is being measured and not nutrient intakes? More detailed description should be added for these measures (could be presented in a table if word count is a concern).<br><br>6. In the Conclusion, the anticipated outcomes and impact of the study could be elaborated (e.g. benefits in terms of interventions). The authors allude to “targetable factors” in the Introduction but some further brief discussion in the Conclusion would be helpful.<br><br>7. The ages of the children have been inconsistently described throughout, switching between months and years (e.g. 48 – 60 months in the abstract and 4 – 5 years in the methods). Consistently describe in months throughout.<br><br>8. Haemoglobin and hemoglobin have inconsistently been used throughout.<br><br>9. Acknowledgments: remove the period in the middle of the first sentence.

**REVIEWER NAME**	***Bosiljka Milosavljevic***
**REVIEWER AFFILIATION**	Queen Mary University of London
**REVIEWER CONFLICT OF INTEREST**	N/A
**DATE REVIEW RETURNED**	28-Nov-2024

**GENERAL COMMENTS**	Thank you for the opportunity to review this manuscript, which describes the methods to be used in The Bahirdar Child Development Study (BCD). Research that explores neural and cognitive development in understudied populations is much needed and this work makes a strong contribution to a growing field. The manuscript itself is clear and well written, there are several suggestions I hope will be helpful to the authors.<br>- The age range of participants in the study is quite large (6mo to 5 yrs). Do you expect to see age-related differences in some of the measures you are implementing? Could you provide some rationale for the age range included?<br><br>Introduction:<br>- While I understand the need to keep the introduction short, it is somewhat too general. The study seems to be focused primarily on acquisition of neuroimaging data and I don't think this is emphasises enough in the introduction. It would be helpful to focus more on why looking at neural development, and brain volume in particular is so important in this context.

	Methods:<br>- Would it be possible to provide more detail on the patient and public involvement? Was the primary goal of this to develop ways to increase acceptability of running MRI scans with children in this community? Or did input from these groups have an impact on the assessments themselves?<br><br>- Study participants and recruitment: Could you indicate what "healthy children" means in this study? As you are recruiting children and infants with potential undernutrition, it would be helpful to specify what the health criteria are.<br><br>- When describing the cognitive and behavioural assessments, it would be helpful if you could indicate whether translation or adaptation was needed. This is mentioned in some places but not for all measures. It would be useful to include a bit more information about how adaptations to existing measures were done.<br><br>- Does the dissemination plan involve communicating some of the research findings to the families involved in this research?<br><br>Thank you again for the opportunity to review this work. I hope the above comments are useful to the authors.

VERSION 1 – AUTHOR RESPONSE

Reviewer: 1

Dr. Taryn Smith, University of Liverpool

Comments to the Author

This protocol describes the Bahirdar Child Development study, a cross-sectional study aiming to characterise the brain and cognitive development of typically developing children spanning 6 – 60 months and factors influencing this development. This study will address gaps in our understanding of biological, environmental and social factors that influence child brain and cognitive development in resource-limited contexts. The authors have clearly described the rationale for the study and data collection methods. Findings from this study are likely to provide insights into a range of factors which influence brain development across a developmental stage of significant brain development (first 5 years) and may point to target and time-sensitive interventions to optimise child development.

Some minor comments and suggestions:

1. In the Study Setting it would be useful to add more information on the setting and the general population of Bahirdar to put the study into context. Similarly, how many language groups are present in the study area and how will this be considered in study recruitment and data collection?

Authors' Response: Thank you for this suggestion. The official language "Amharic" is spoken in the study area. Thus, the consent form and tools were translated into Amharic to be used for data collection. We have added extra descriptions of the setting and general population in the "Study Setting" section.

"Study setting (Page 5, line 13)

Bahir Dar is the capital city of the Amhara Region in Ethiopia where the official language is Amharic. The population of Bahir Dar was projected to be 348,429 in 2017 [8]. According to the 2023 United Nations Development Programme multidimensional poverty index, 27% of the Ethiopian Population lives below the US $2.15 per day income poverty line [9]. According to the 2019 Ethiopian Demographic and Health Survey report, 49.8% of women aged 15-49 years in the Amhara region are literate [10]. Moreover, the rate of stunting among children under five years of age is 41.5% [10]. The study will be conducted in two health centers in Bahirdar (Shimbit and Abay Mado) and Felege Hiwot Comprehensive Specialized Hospital (FHCSH). FHCSH is the main referral hospital in the region and delivers health care services for more than 10 million people in Amhara and neighbouring regions. A Hyperfine low-field (0.064 Tesla) MRI machine was placed in FHCSH on June 18, 2022 and the installation was completed on September 1, 2022."

2. Table 2, final bullet point under the inclusion criteria, is there some age bands 24 – 60 months) missing at the end of the sentence?

Authors' Response: Thank you for bringing this to our attention. Part of the sentence was indeed missing and we have edited Table 1 accordingly. It now reads:

Table 1: Inclusion and exclusion criteria and additional MRI screening (page 7)

"The child is within specified age bins for one of the cohort/group +/- 1 months for the 6, 12, and 18 months old and +/- 2 months for the 24, 36, 48, and 60 months old children."

3. A rationale for why the specific child development measures (visual reasoning, verbal expression, school readiness, executive functions) were selected should be briefly added.

Authors' Response: Thank you for this suggestion. Based on your comment, we made the edits to the Introduction and the Methods section:

Introduction (Page 5, line 31)

[...] "We focus on developmental and neurocognitive outcomes that reflect early developmental milestones in children aged 6 to 36 months. In older children aged 48 and 60 months, we assess domains that we believe contribute to school readiness including aspects of visual reasoning, attention, processing speed, verbal development and early literacy, numeracy, and early knowledge."

Methods; Other measures (Page 10)

“All children will undergo an anthropometric assessment and a cognitive developmental assessment. Children aged 6-36 months will complete the GSED while children aged 48 and 60 months will complete a battery of tasks selected to assess different aspects of cognitive development, executive functions, and self-regulation as well as a tool to assess early math skills, literacy, and knowledge as all of these domains are believed to reflect aspects of child-level school readiness. Tools were piloted in March 2024 and minor adaptations were made to be culturally relevant, feasible, acceptable, and age appropriate.”

We have also made edits to the sections describing individual tools to provide more context for why each tool was selected.

4. The authors state that adaptations to the child development tools have been made. It would be helpful to describe these adaptations briefly.

Authors’ Response: Thank you for this suggestion. The only standardized tool that we adapted was the GSED which we translated and adapted in close collaboration with WHO. We have added a brief description of the process under the WHO GSED section on page 10-11. The adaptation process followed a process whereby it was translated into Amharic language by two independent early childhood experts who held a consensus meeting with local early child development field experts to make sure the tool was culturally appropriate. These adaptations were then back-translated, then approved by WHO, and piloted. A separate paper on the GSED adaptations and implementation is currently under review.

The verbal reasoning task, matrix reasoning task, and the school readiness questionnaires had already been adapted to the region by other researchers who shared the tool, and we made only minor adaptations based on feedback from children and experts during pre-study piloting. Most of these changes concerned drawn images that the child did not recognize; for example, for one drawn image of a lake picture we selected a higher quality more recognizable image (See page 11).

5. I find the surveys for the key exposures inadequately described (Table 4). For example, PHQ-9 is neither defined nor described. How is nutrient intake being estimated? Or is it simply dietary diversity that is being measured and not nutrient intakes? More detailed description should be added for these measures (could be presented in a table if word count is a concern).

Authors’ Response: We cut this content due to word count constraints but have added descriptions to Table 4 as suggested.

6. In the Conclusion, the anticipated outcomes and impact of the study could be elaborated (e.g. benefits in terms of interventions). The authors allude to “targetable factors” in the Introduction but some further brief discussion in the Conclusion would be helpful.

We added more reflection on the planned analyses to the Conclusion

CONCLUSION (page 16, line 1)

“By cross-sectionally assessing children across seven age categories, we will map stages of neurological development in a semi-rural population in sub-Saharan Africa. The study was designed to be harmonised with similar ongoing cohort studies collecting MRI in paediatric populations globally [12]. The combined assessment of neurocognitive and MRI outcomes will allow us to examine possible associations between neuroanatomical outcomes and neurocognition. Using questionnaires to characterise children’s biological and psychosocial experiences we will be able to assess associations between neurocognitive outcomes and environmental experiences. Analyses will examine correlations between neuroanatomical outcomes and cognitive/ behavioural outcomes as well as associations of adverse risk factors (e.g. low socioeconomic status and poverty, food insecurity, stunted growth, poor maternal mental health, high levels of hardship) and protective factors (e.g. stimulating home environments) with neuroanatomical and cognitive / behavioural outcomes. Few studies have implemented MRI in semi-rural settings in LMICs. This study will generate data to guide studies and future interventions for traditionally hard-to-reach and under-represented research populations”

7. The ages of the children have been inconsistently described throughout, switching between months and years (e.g. 48 – 60 months in the abstract and 4 – 5 years in the methods). Consistently describe in months throughout.

Authors’ Response: Thank you for sharing this observation. This has been fixed to months.

8. Haemoglobin and hemoglobin have inconsistently been used throughout.

Authors’ Response: Thank you. This has been fixed to be consistent as “haemoglobin”.

9. Acknowledgments: remove the period in the middle of the first sentence.

Authors’ Response: Thank you, this has been fixed.

Reviewer: 2

Bosiljka Milosavljevic, Queen Mary University of London

Comments to the Author

Thank you for the opportunity to review this manuscript, which describes the methods to be used in The Bahirdar Child Development Study (BCD). Research that explores neural and cognitive development in understudied populations is much needed and this work makes a strong contribution to a growing field. The manuscript itself is clear and well written, there are several suggestions I hope will be helpful to the authors.

1. The age range of participants in the study is quite large (6mo to 5 yrs). Do you expect to see age-related differences in some of the measures you are implementing? Could you provide some rationale for the age range included?

Authors' Response: The age range was selected to cross-sectionally characterize associations of MRI volume metrics with behavioral outcomes and between risks and MRI volumes and behavior. We consider the study to be exploratory with regard to differences across the age spans. Still, we would expect to see stronger correlations between volumes and behavior in older children and similarly stronger associations between risk exposure and neuro-cognitive outcomes in older children. This would be in line with observations from other neuroimaging studies in LMIC, such as the BEAN study in Bangladesh.

We have added goals and hypotheses to the Introduction (page 3-4).

[...]"The first goal of the study is to examine correlations between brain volumes and neurocognitive / developmental outcomes. The second goal is to examine associations between risk and protective factors and neurodevelopmental outcomes. We will explore these relationships across different age groups. We focus on developmental and neurocognitive outcomes that reflect early developmental milestones in children aged 6 to 36 months. In older children aged 48 and 60 months, we assess domains that we believe contribute to school readiness including aspects of visual reasoning, attention, processing speed, verbal development and early literacy, numeracy, and early knowledge. Previous studies in LMIC have shown differences in associations of early life exposures and neurodevelopmental outcomes [7]"

Introduction:

2. While I understand the need to keep the introduction short, it is somewhat too general. The study seems to be focused primarily on acquisition of neuroimaging data and I don't think this is emphasises enough in the introduction. It would be helpful to focus more on why looking at neural development, and brain volume in particular is so important in this context.

Authors' Response: Thank you for this comment. The acquisition of neuroimaging data was a goal, but the broader goals of the study extend beyond a feasibility study. Specifically, the study aims to examine associations between brain volumes and behavioral outcomes and between brain outcomes, behavioral outcomes, and risk and protective factors. As mentioned above we have added more explicit content on these goals. Total supratentorial brain volume was picked as crude neuroanatomical outcomes based on

consultation with groups implementing similar hyperfine MRI methods in similar settings. Of note, the image resolution is low so we needed to define a realistic primary MRI outcome.

Methods:

3. Would it be possible to provide more detail on the patient and public involvement? Was the primary goal of this to develop ways to increase acceptability of running MRI scans with children in this community? Or did input from these groups have an impact on the assessments themselves?

Authors' Response: As part of a larger study now cited in the Methods section, we conducted in-depth interviews with community members; mothers, fathers, and community leaders (n=24) to assess the acceptability of MRI scans in children after watching explanatory short videos. It was found that even though their knowledge about MRI was limited, they responded positively to the informative video and were found acceptable. The informative video was revised based on the in-depth interviews. Experiences from this work will be presented in a separate publication.

4. Study participants and recruitment: Could you indicate what "healthy children" means in this study? As you are recruiting children and infants with potential undernutrition, it would be helpful to specify what the health criteria are.

Authors' Response: Thank you for this important clarifying point. More description has been added.

The criteria are listed in the exclusion criteria (Table 1) but are now also highlighted in the text

"Study participants and recruitment

The BCD study will enroll healthy children from age 6 through 60 months across eight cohorts (see Table 1). Eligible children will be identified during routine outpatient and vaccination visits at the study health centers. Children who present with clinical symptoms based on parental/ child self-report will not be eligible to participate. We will aim to recruit N=50 children into each cohort of the eight cohorts for a total cross-sectional sample size of N=400. Inclusion and exclusion criteria and additional MRI screening questions are shown in Table 2."

5. When describing the cognitive and behavioural assessments, it would be helpful if you could indicate whether translation or adaptation was needed. This is mentioned in some places but not for all measures. It would be useful to include a bit more information about how adaptations to existing measures were done.

Authors' Response: Thank you for raising this important point. We also discuss this under question number 4 above. The only standardized tool that we adapted was the GSED, which we translated and adapted in close collaboration with WHO. We have added a brief description of the process under the

WHO GSED section. The verbal reasoning task, matrix reasoning task, and the school readiness questionnaires had already been adapted to the region by other researchers who shared the tool and we made only minor adaptations based on feedback from children and experts during pre-study piloting. We have added more details about adaptations.

6. Does the dissemination plan involve communicating some of the research findings to the families involved in this research?

Authors' Response: Families were notified of assessment results individually when appropriate. The overall study results will be communicated to the health centers and local health authorities.

Thank you again for the opportunity to review this work. I hope the above comments are useful to the authors.

Authors' Response: Thank you for this helpful feedback.

VERSION 2 – REVIEW

**REVIEWER NAME**	***Taryn Smith***
**REVIEWER AFFILIATION**	University of Liverpool
**REVIEWER CONFLICT OF INTEREST**	N/A
**DATE REVIEW RETURNED**	10-Feb-2025

**GENERAL COMMENTS**	Thank you for comprehensively addressing my minor suggestions. I have no further comments.

**REVIEWER NAME**	***Bosiljka Milosavljevic***
**REVIEWER AFFILIATION**	Queen Mary University of London
**REVIEWER CONFLICT OF INTEREST**	N/A
**DATE REVIEW RETURNED**	21-Feb-2025

**GENERAL COMMENTS**	Thank you for the opportunity to review this revised manuscript. The authors have been very receptive to the reviewers' suggestions and the manuscript is in good shape now.
